# Swift tuning from spherical molybdenum microspheres to hierarchical molybdenum disulfide nanostructures by switching from solvothermal to hydrothermal synthesis route

**DOI:** 10.1186/s40580-017-0119-9

**Published:** 2017-09-29

**Authors:** Nilam Qureshi, Sudhir Arbuj, Manish Shinde, Sunit Rane, Milind Kulkarni, Dinesh Amalnerkar, Haiwon Lee

**Affiliations:** 1 Centre for Materials for Electronics Technology (C-MET), Panchwati Off Pashan Road, Pune, 411008 India; 2 Institute of Nano Science and Technology, Hanyang University, Seoul, 04763 Republic of Korea; 3 Department of Chemistry, Hanyang University, Seoul, 04763 Republic of Korea

**Keywords:** Metallic molybdenum, Molybdenum disulfide, Microsphere, Hierarchical nanostructures, Solvothermal, Hydrothermal

## Abstract

Herein, we report the synthesis of metallic molybdenum microspheres and hierarchical MoS_2_ nanostructures by facile template-free solvothermal and hydrothermal approach, respectively. The morphological transition of the Mo microspheres to hierarchical MoS_2_ nanoflower architectures is observed to be accomplished with change in solvent from ethylenediamine to water. The resultant marigold flower-like MoS_2_ nanostructures are few layers thick with poor crystallinity while spherical ball-like molybdenum microspheres exhibit better crystalline nature. This is the first report pertaining to the synthesis of Mo microspheres and MoS_2_ nanoflowers without using any surfactant, template or substrate in hydro/solvothermal regime. It is opined that such nanoarchitectures of MoS_2_ are useful candidates for energy related applications such as hydrogen evolution reaction, Li ion battery and pseudocapacitors. Inquisitively, metallic Mo can potentially act as catalyst as well as fairly economical Surface Enhanced Raman Spectroscopy (SERS) substrate in biosensor applications.

## Background

In the family of molybdenum compounds, metallic molybdenum and molybdenum disulfide are the least and the most explored entities as far as their synthesis is concerned. Curiously, research on molybdenum nanoparticles has not been adequately pursued as compared to the noble metals such as gold and silver though it is cheaper and readily available and has the potential of being used in catalytic, refractory plasmonic as well as in biosensing applications. There are sporadic reports pertaining to the synthesis of molybdenum nanostructures [1–8]. Mostly, molybdenum nanostructures have been generated by various plasma techniques such as RF plasma [1], DC magnetron sputtering [2, 3], electron cyclotron resonance plasma [4] etc. Recently, nanocomposites of Mo-polyphenylene sulfide (PPS) and Mo-MoO_3_-PPS have also been reported by novel polymer-inorganic solid-state reaction method [9]. However, synthesis of molybdenum nanostructures using facile solvothermal route has not been reported so far.

Molybdenum sulfide is an exotic class of materials exhibiting very interesting properties in its 0D, 1D, 2D and 3D forms [10–14]. Among different molybdenum sulfide compounds, molybdenum disulfide (MoS_2_) is the most important and is being researched fundamentally, computationally and experimentally across the globe. Especially, hierarchical nanostructures of MoS_2_ are very important from the standpoint of their applications in diverse fields revolving around biology and electronics as they simultaneously possess bulk nature due to a bigger size as well as quantum confinement effects due to nanosheet-like nature of petals. Owing to such attributes, MoS_2_ nanoflowers have been produced using different methods such as hydro/solvothermal route, sol–gel route, chemical process, chemical vapor deposition, etc. [15–22]. There are some reports pertaining to hydrothermal synthesis of MoS_2_ nanostructures with flower-like morphology for vivid applications [11, 17, 18, 23–26]. Herein, we present simple one-pot protocol dealing with synthesis of metallic molybdenum microspheres and hierarchical MoS_2_ nanostructures by reasonably scalable solvothermal and hydrothermal technique, respectively, just by imparting a change of solvent.

## Experimental details

All chemicals were of reagent grade and were used as received. The synthesis of molybdenum disulfide was carried out through hydrothermal route. In typical procedure, 1 mM of ammonium heptamolybdate tetrahydrate was added in 30 ml of deionized water (DIW) in a beaker and stirred for 20 min till it is dissolved. Similarly, in another beaker, 3 mM of thiourea was taken in 30 ml of DIW and stirred for 20 min with final molar ratio of Mo:S precursor being 1:3. Both the solutions were mixed and subjected to rigorous stirring for another 20 min. The resultant solution was then transferred into 100 ml Teflon-lined stainless-steel autoclave which was properly sealed and placed in an oven for hydrothermal treatment at 200 °C for 9 h. The oven is allowed to cool naturally to room temperature. Subsequently, the resulting black solid was retrieved from the solution by centrifugation, washed with distilled water followed by ethanol two times to remove the ions possibly remaining in the end product, and finally dried at 60 °C for 6 h, respectively.

Similar reaction was repeated in another set of experiment using mixture of ethylenediamine and DIW in 5:1 volume ratio (solvothermal route) keeping all other experimental conditions the same. The samples prepared corresponding to the hydrothermal and solvothermal routes are labeled as MS9 and Mo9, respectively. The structural information on virgin powder samples was obtained using X-ray diffraction (Bruker D8 Advance) technique. The diffraction angle 2(θ) was varied between 10 and 80° range and the observed XRD peaks were compared with standard JCPDS cards. The surface morphological features of the samples were investigated by field emission scanning electron microscopy (FESEM) using HITACHI S-4800. The powder sample was directly placed on the conducting carbon film and was coated with a thin Au–Pd film by sputtering to avoid the effects due to charging of the sample. The fine-scale microstructure of the samples was examined by field emission transmission electron microscopy (FETEM) with JEM-2200FS (JEOL, Japan), at an acceleration voltage of 200 kV. The samples for FETEM were prepared by dispersing fine powder of the resultant product in isopropyl alcohol. A drop of dispersion was then transferred to carbon coated grid for further analysis. The Brunauer–Emmett–Teller (BET) surface area measurements were performed using a NOVAtouch LX^1^ surface area and pore size analyzer from Quantachrome Instruments, USA.

## Results and discussion

**Fig. 1:**
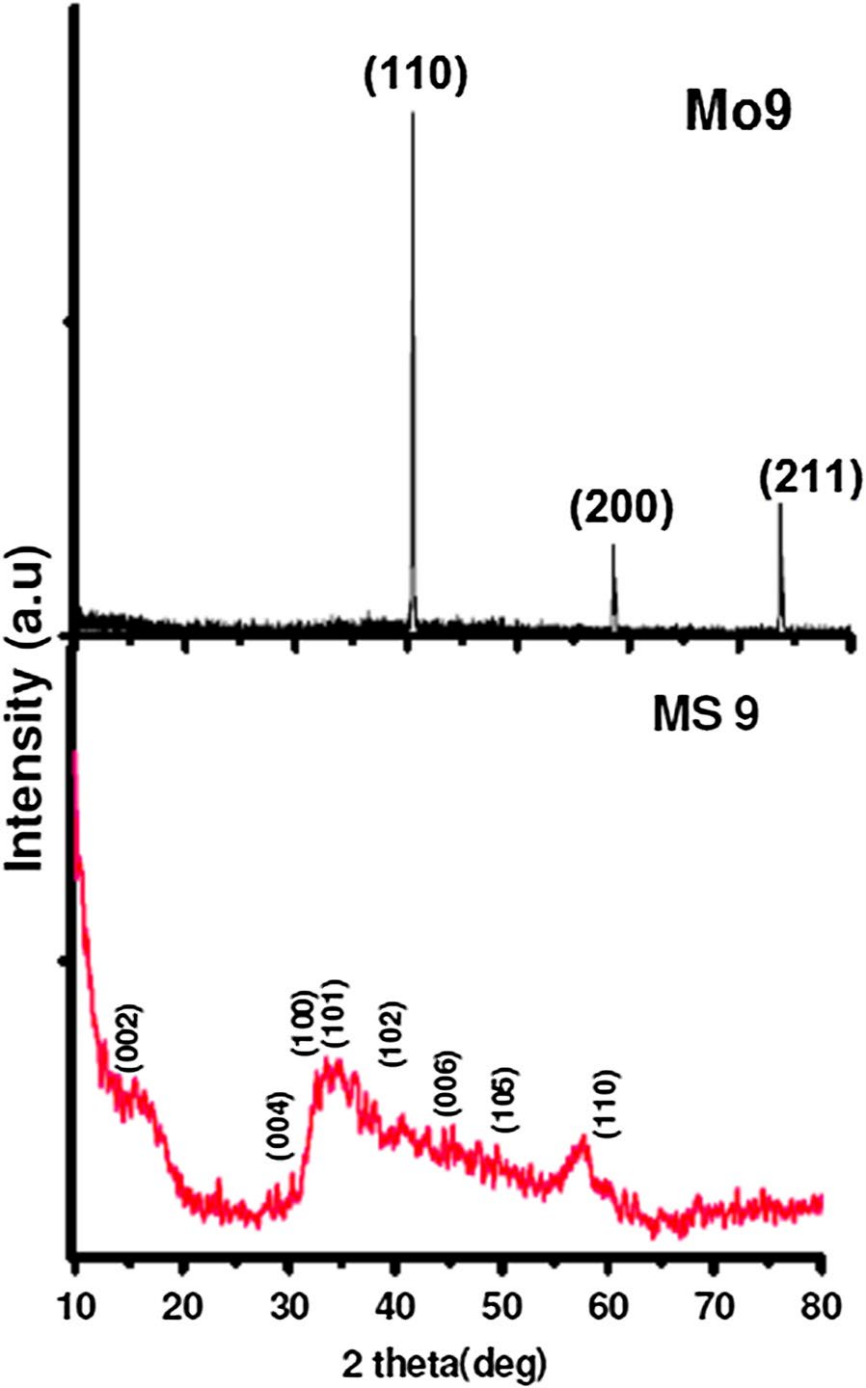
X-ray diffractograms of the resultant powder samples, Mo9 and MS9

The X-ray diffractograms of the as-synthesized powder samples are reproduced in Fig. 1. In case of Mo9 sample, very sharp peaks at 2θ values of 40.54°, 58.57° and 73.56° are seen which can be indexed to the (110), (200) and (211) planes of face-centered cubic structure of the metallic molybdenum (JCPDS# 040809). Very sharp peaks indicate the formation of highly crystalline and bigger particles. On the contrary, in case of MS9 sample, two broad peaks centered around 2θ ~33° and 57° are observed. These can be correlated with the relatively low-intensity peaks at 32.89°, 33.4° & 35.3° and 56.2° and 58.3°, respectively, which can be matched well with MoS_2_ phase (JCPDS card# 37-1492). Significant peak broadening observed for this sample corresponding to hydrothermal synthesis protocol can be credited to (a) smaller crystallite size and (b) layered nature of hexagonal MoS_2_. The unresolved peak at 2θ ~15° which is ordinarily attributable to (002) plane (maximum intensity peak in the standard pattern of bulk 2H-MoS_2_) and the diffused background are indicative of random stacking of MoS_2_ layers and the consequent disordered packing. In this context, it can be speculated that during the hydrothermal reaction between ammonium molybdate and thiourea, both the precursors release ammonia which might affect the stacking of MoS_2_ nanostructures at some step of the reaction path [26]. Nevertheless, because of the presence of characteristic peaks both at ~33° and 57°, it can be inferred that MoS_2_ is the dominant phase with no extraneous phase in this sample. Thus, transition of reaction product from metallic molybdenum to semiconducting MoS_2_ is observed with change in solvent from ethylenediamine (solvothermal) to water (hydrothermal) in the synthesis protocol.

**Fig. 2:**
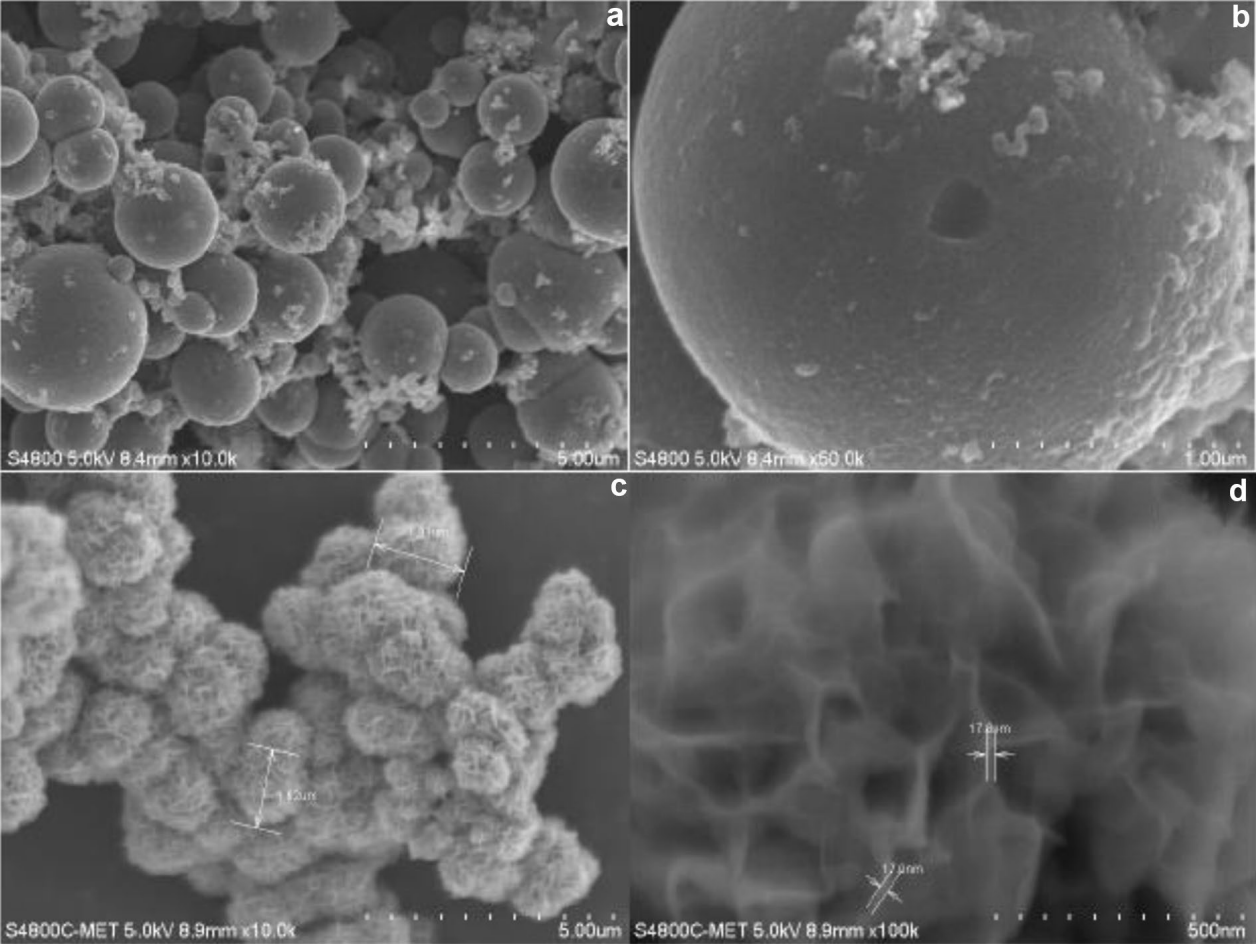
FESEM images of the sample Mo9 under **a** low-magnification **b** high-magnification and sample MS9 under **c** low-magnification and **d** high-magnification

FESEM images for the resultant powders are displayed in Fig. 2a–d. In Fig. 2a, the different spherical ball-like microspheres of metallic molybdenum with diverse size ranging from 2 μm to 500 nm are observed. However, the average size of these microspheres is around 1.5 μm. FESEM images of the sample corresponding to hydrothermal route (MS9) illustrate the formation of hierarchical molybdenum sulfide nanoflowers. The low magnification FESEM image (Fig. 2c) of the sample MS9 displays hierarchical marigold flower-like structures having a size in the range of 1 to 2 µm. At high magnification, as seen in Fig. 2d, each marigold flower appears to be made up of petal-like nanostructures [27, 28]. Each petal has thickness ~10–20 nm. Thus, we can observe totally different morphologies corresponding to samples Mo9 and MS9. It can be apparently attributed to the prevailing thermodynamic conditions associated with the change in solvent which, in turn, govern the nucleation and growth steps in the individual solvent-controlled high pressure reactions.

**Fig. 3:**
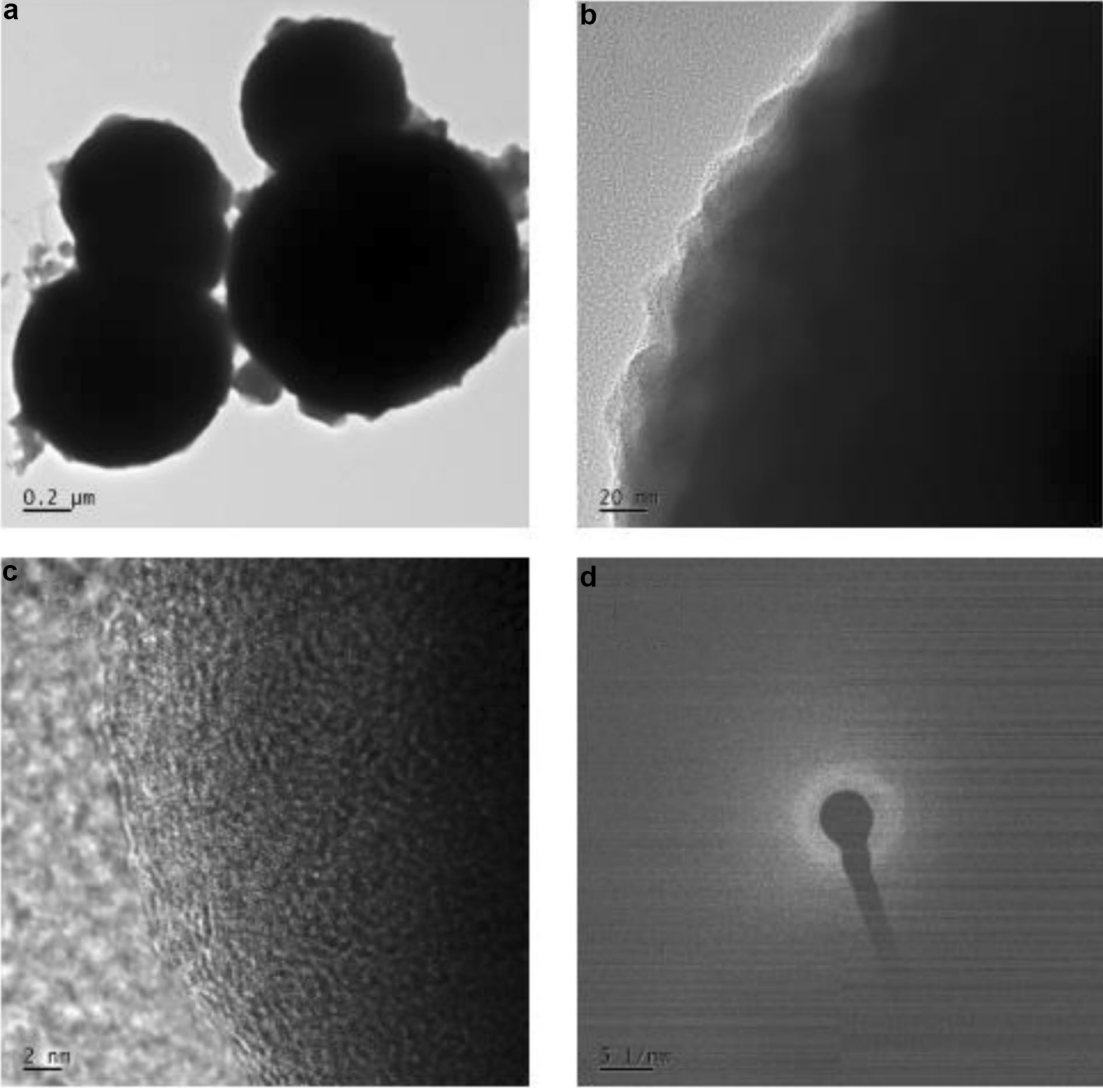
HRTEM images of the typical Mo9 sample **a** low-magnification, **b** high-magnification, **c** lattice image and **d** SAED pattern

**Fig. 4:**
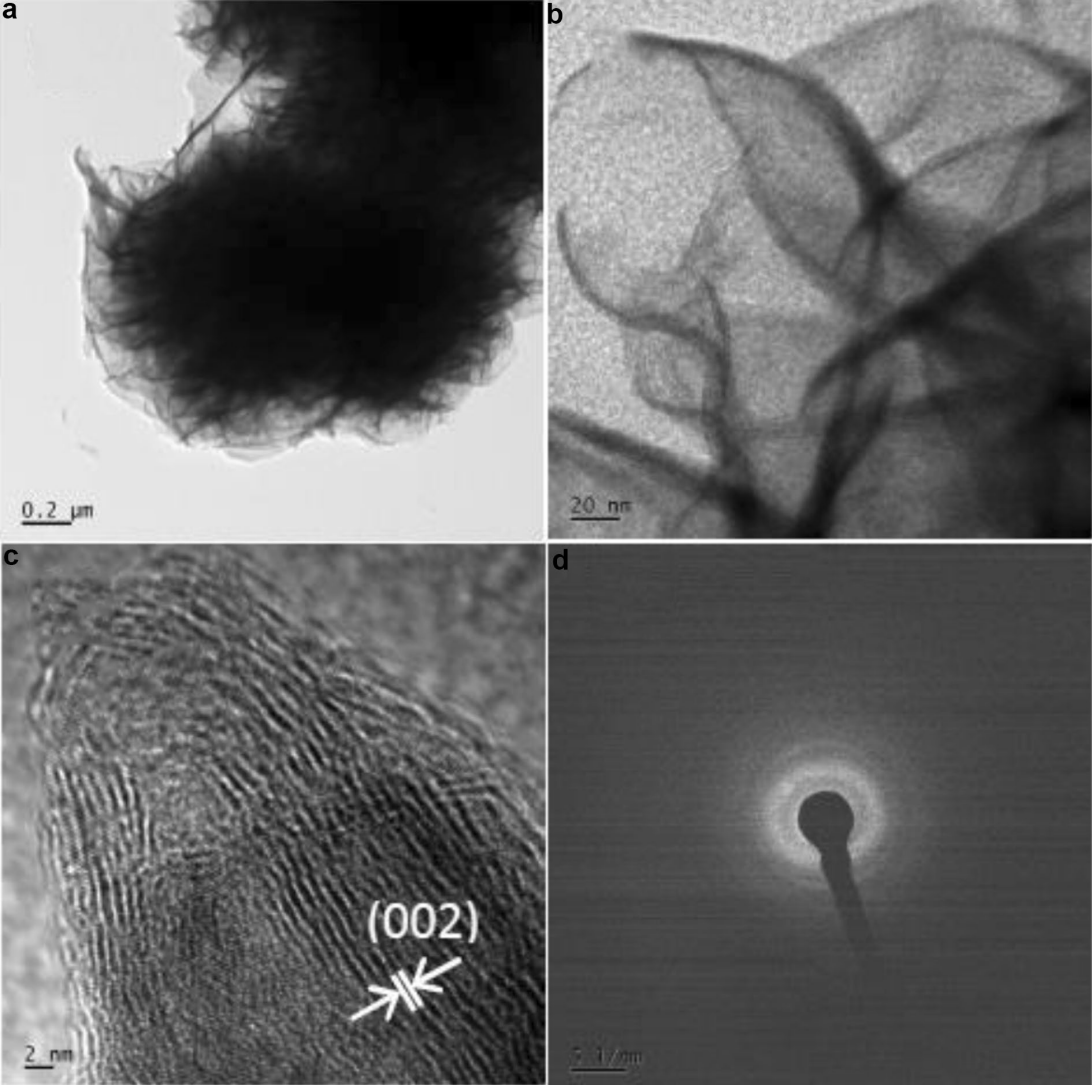
HRTEM images of the typical MS9 sample **a** low-magnification, **b** high-magnification, **c** lattice image and **d** SAED pattern

To ascertain the fine-scale microstructure of the resultant powder, representative FETEM images of the samples Mo9 and MS9 were obtained as shown in Figs. 3 and 4, respectively. In the case of the sample prepared via solvothermal route (Mo9), the low-magnification TEM image reveals the formation of spherical ball-like microsphere structures (Fig. 3a). However, from high-magnification image (Fig. 3b), it appears that the edges of spherical ball-like microspheres are thicker and appeared to be made up of multiple layers. These spherical ball-like microsphere structures correlate well with the similar structures seen in FE-SEM images for this sample. SAED pattern reveals halo ring-like patterns indicating amorphous nature of the sample.

The TEM images disclose the predominant formation of flower-like nanostructures in the case of MS9 sample (Fig. 4a). At higher-magnification, we could observe that the petals, which are fragile in appearance, encompass few layers of MoS_2_ (Fig. 4b). The lattice image (Fig. 4c) indicates that the petal at the twist is made up of around 20 layers with the d value as 6.2 Å which matches with the available reports of few layered thick MoS_2_ nanostructures [10]. The BET surface area for Mo and MoS_2_ nanostructures was calculated to be 2.31 and 18.62 m^2^/g, respectively. Higher surface area observed in case of MoS_2_ nanoflowers is attributable to its flower like morphology.

## Conclusions

Synthesis of hierarchical nanostructures of MoS_2_ has been accomplished by simple hydrothermal route without resort to usage of surfactant/substrate/template while the solvothermal route using ethylenediamine as solvent leads to formation of metallic Mo microspheres under similar reaction conditions. Direct oxidation of ammonium molybdate to Mo in presence of thiourea via solvothermal route is a salient aspect of our work which needs new understanding of super-critical conditions controlled chemical reactions. The obtained hierarchical nanostructures of MoS_2_ possess very high surface area due to the presence of petal-like surface features. Such high surface area in MoS_2_ nanostructures along with lesser degree of resistance due to stacking layers can be useful for applications in hydrogen evolution reaction (HER) and pseudocapacitors. While metallic Mo can be explored as novel catalyst and possibly as the economical SERS substrate in biosensor and refractory plasmonic applications, it may be noted that the relevant literature data on such studies is not readily available to the best of our knowledge. Therefore, our efforts in these application-oriented directions are underway.
